# Gene expression analysis of the biocontrol fungus *Trichoderma harzianum *in the presence of tomato plants, chitin, or glucose using a high-density oligonucleotide microarray

**DOI:** 10.1186/1471-2180-9-217

**Published:** 2009-10-13

**Authors:** Ilanit Samolski, Alberto de Luis, Juan Antonio Vizcaíno, Enrique Monte, M Belén Suárez

**Affiliations:** 1 Centro Hispano-Luso de Investigaciones Agrarias (CIALE), Universidad de Salamanca. Campus de Villamayor-Parque Científico, 37185 Villamayor, Salamanca, Spain; 2 Centro de Investigación Biomédica de La Rioja (CIBIR). Piqueras 98, 26006 Logroño, La Rioja, Spain; 3 EMBL Outstation, European Bioinformatics Institute, Wellcome Trust Genome Campus, Hinxton, Cambridge, UK; 4 Current address: Instituto de Microbiología Bioquímica, CSIC/Universidad de Salamanca. Campus Miguel de Unamuno, 37007 Salamanca, Spain

## Abstract

**Background:**

It has recently been shown that the *Trichoderma *fungal species used for biocontrol of plant diseases are capable of interacting with plant roots directly, behaving as symbiotic microorganisms. With a view to providing further information at transcriptomic level about the early response of *Trichoderma *to a host plant, we developed a high-density oligonucleotide (HDO) microarray encompassing 14,081 Expressed Sequence Tag (EST)-based transcripts from eight *Trichoderma *spp. and 9,121 genome-derived transcripts of *T. reesei*, and we have used this microarray to examine the gene expression of *T. harzianum *either alone or in the presence of tomato plants, chitin, or glucose.

**Results:**

Global microarray analysis revealed 1,617 probe sets showing differential expression in *T. harzianum *mycelia under at least one of the culture conditions tested as compared with one another. Hierarchical clustering and heat map representation showed that the expression patterns obtained in glucose medium clustered separately from the expression patterns observed in the presence of tomato plants and chitin. Annotations using the Blast2GO suite identified 85 of the 257 transcripts whose probe sets afforded up-regulated expression in response to tomato plants. Some of these transcripts were predicted to encode proteins related to *Trichoderma*-host (fungus or plant) associations, such as Sm1/Elp1 protein, proteases P6281 and PRA1, enchochitinase CHIT42, or QID74 protein, although previously uncharacterized genes were also identified, including those responsible for the possible biosynthesis of nitric oxide, xenobiotic detoxification, mycelium development, or those related to the formation of infection structures in plant tissues.

**Conclusion:**

The effectiveness of the *Trichoderma *HDO microarray to detect different gene responses under different growth conditions in the fungus *T. harzianum *strongly indicates that this tool should be useful for further assays that include different stages of plant colonization, as well as for expression studies in other *Trichoderma *spp. represented on it. Using this microarray, we have been able to define a number of genes probably involved in the transcriptional response of *T. harzianum *within the first hours of contact with tomato plant roots, which may provide new insights into the mechanisms and roles of this fungus in the *Trichoderma*-plant interaction.

## Background

The ability of some fungal species of the genus *Trichoderma *to suppress disease and stimulate the growth and development of plants explains the wide and long-term use of these organisms in many crops [1]. Traditionally, the beneficial effects of *Trichoderma *spp. on plants have been attributed to their capability to antagonize soil-borne pathogens by a combination of mycoparasitism, secretion of antibiotics, and competition for space and substrates [2]. However, subsequent discoveries have demonstrated that these biocontrol agents are also able to interact intimately with plant roots, even colonizing the outer epidermis layers, and to act as opportunistic, avirulent plant symbionts [3]. Currently, it is known that the root colonization by *Trichoderma *spp. produces changes in plant metabolism that can lead to increased root development, crop productivity, and resistance to abiotic and biotic stresses [4].

In recent years, increased attention has been paid to studying the direct interactions occurring between *Trichoderma *spp. and plants, including molecular studies of specific bioactive components produced by the fungal partner that have been associated with plant defence mechanism elicitation, root colonization, or plant growth promotion [5-12]. Novel genomic and proteomic techniques are also now being implemented to *Trichoderma *biocontrol species with the aim of identifying large-scale molecular factors involved in the communication between *Trichoderma *and plants. Macroarray analyses have been applied to study the gene expression of four species of *Trichoderma *during their interaction with cacao seedlings [13], and of *T. harzianum *during the early colonization of tomato roots [14]. There is also a study based on a three-way interaction system (bean plant-pathogen-*T. atroviride*) that used a proteomic approach to identify differential proteins produced by each of the three organisms involved in that association [15]. Apart from this, several recent works on plant-*Trichoderma *interactions have been conducted to explore the molecular responses of plants to the presence of a root-colonizing *Trichoderma *strain, using either transcriptomic [16] or proteomic methods [17,18].

Microarray analyses are becoming a powerful tool for large-scale gene expression studies in filamentous fungi [19]. However, transcriptomic analyses of *Trichoderma *biocontrol species using this technology have been hampered by the scant sequencing conducted on these fungi. In fact, the first analysis of the genome sequence of a Trichoderma strain (T. reesei QM 6a) has been recently published [20], although this sequence has been publicly available for a few years. Fortunately, the first version of the complete genome from two other Trichoderma species, the biocontrol agents T. virens Gv29-8 and T. atroviride IMI 206040, is now available on-line [21,22]. Since the complete genomes of other *Trichoderma *biocontrol species are not available and nor will they be in the near future, in this work we focused our efforts on developing a customized high-density oligonucleotide (HDO) microarray from a large Expressed Sequence Tag (EST) collection, which was generated in a previous EU-funded project called "TrichoEST" [23-25]. This project has provided a fundamental resource for transcriptomic analyses in *Trichoderma *spp. through the sequencing of more than 25,000 ESTs from eight different species representing the biodiversity of this genus: *T. harzianum*, *T. atroviride, T. asperellum, T. viride, T. longibrachiatum, T. virens, T. stromaticum *and *T. aggresivum*. Specifically, these ESTs were obtained from 28 cDNA libraries under a wide range of growth conditions, including biocontrol-related conditions and different nutritional situations [23-25].

The aim of the present study was to explore transcriptomic changes in the biocontrol strain *T. harzianum *CECT 2413 in its early interactions with tomato plant roots using microarray technology. We report the construction of a *Trichoderma *HDO microarray composed of 384,659 25-mer probes designed against 14,081 EST-derived transcripts from twelve strains belonging to the eight *Trichoderma *species cited above, and 9,121 genome-derived transcripts from *T. reseei *[20], since it was the only entire *Trichoderma *genome available when the microarray was designed. As far as we know, this is the first time that an oligonucleotide microarray has been used to study gene expression changes of a *Trichoderma *strain in the presence of a plant host. RNAs from *T. harzianum *CECT 2413 mycelia cultured in the presence and absence of tomato plants and also in glucose- or chitin-containing media were hybridized to the *Trichoderma *HDO microarray proposed in this work.

## Results

### *Trichoderma *HDO microarray design

**Figure 1:**
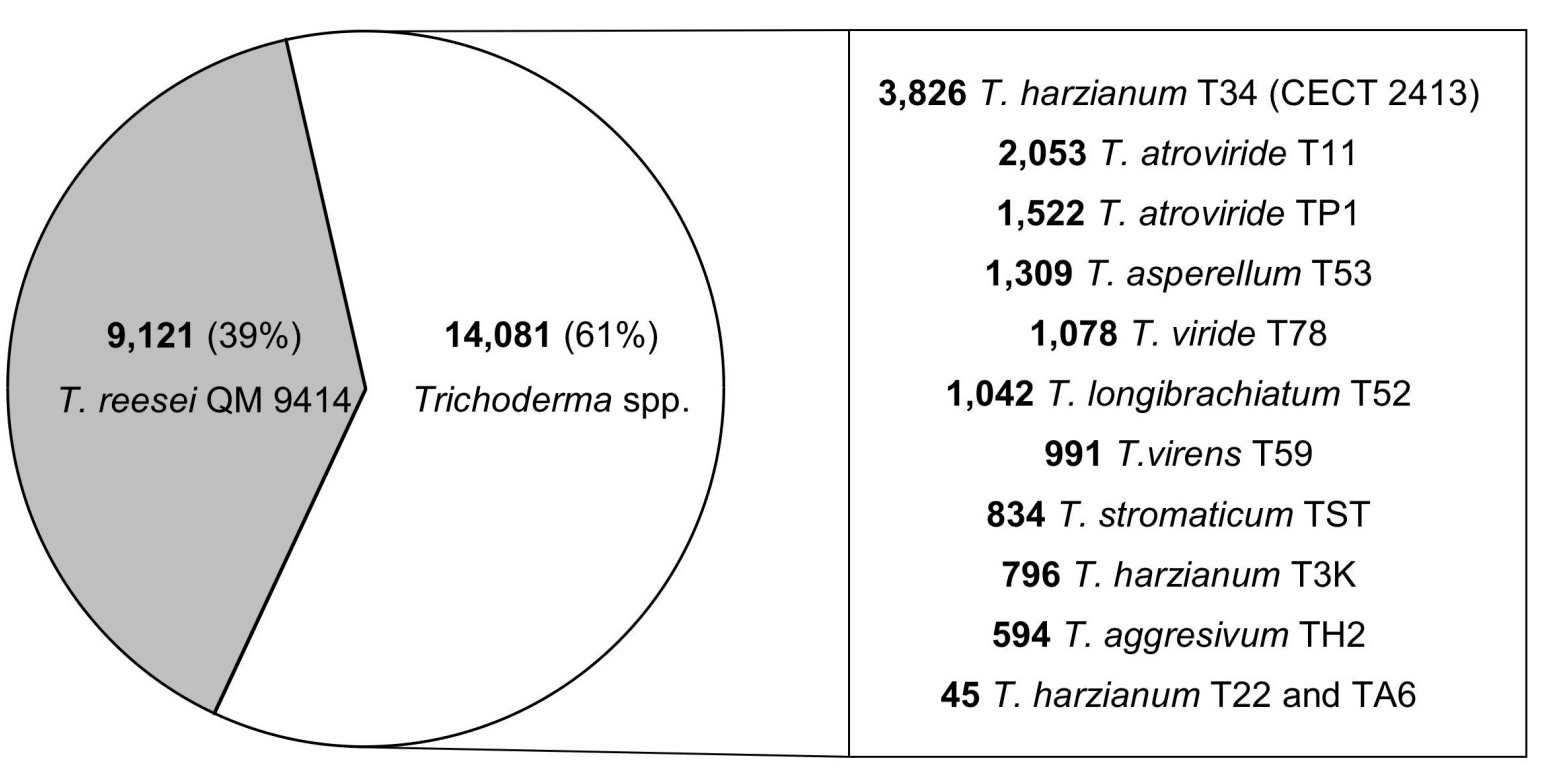
***Trichoderma *HDO microarray composition**. Number of gene transcripts of *Trichoderma *spp. (EST-derived) and *T. reesei *(genome-derived) represented on the *Trichoderma *HDO microarray generated in the present work.

The probe selection process conducted as described in Methods yielded a total of 384,659 different probes [GEO accession number: GPL7702] that were included on our custom-designed *Trichoderma *HDO microarray. After mapping these individual probes to the initial collections of EST-derived transcripts of twelve *Trichoderma *strains and genome-derived transcripts of *T. reesei*, from which the probes were designed, it was found that approximately 35% of the probes on the chip matched transcripts from *Trichoderma *spp. and about 65% matched transcripts from *T. reesei*, which was consistent with the size in base-pairs of each of the two sequence collections (7.1 and 13.9 Mbp, respectively). Moreover, 1.5% of the probes on the chip could be mapped to sequences from both databases. The number of probes associated with each particular transcript sequence (probe set size) ranged from 1 to 94 for *Trichoderma *spp. transcripts, and from 1 to 1,245 for *T. reesei *transcripts, with a median value of 16 and 22, respectively, and a maximum of approximately 40 nt between adjacent probes (data not shown). The final composition of the microarray in terms of the number of transcript sequences of each *Trichoderma *strain represented by a probe set is shown in Figure 1. In all, of the original 14,237 EST-derived sequences of *Trichoderma *spp. and 9,129 genome-derived sequences of *T. reesei*, only 156 (1,1%) and 8 (0.1%), respectively, were not represented on the microarray since no probe passed the selection procedure (the identification codes of the excluded sequences are available as supplementary material in additional file 1).

### Overview of expression data in *T. harzianum *from microarray analysis

**Figure 2:**
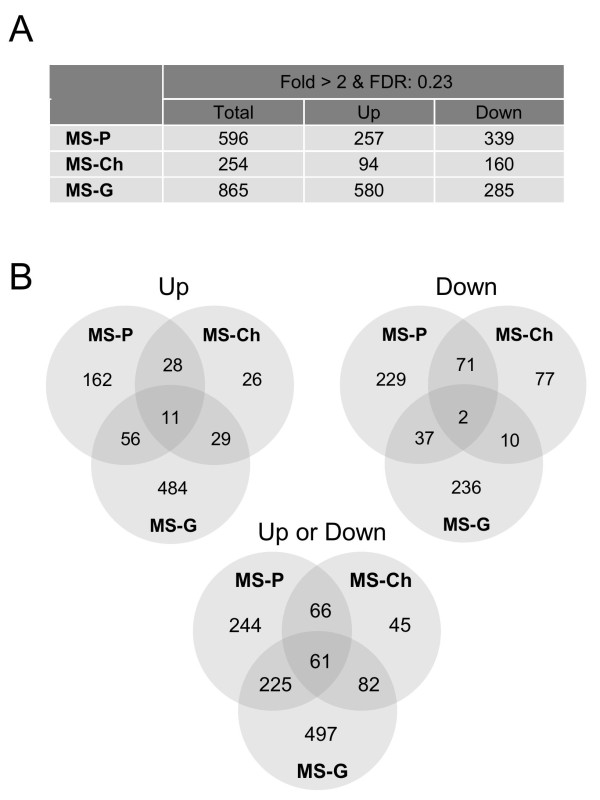
**Global expression data in *T. harzianum *from microarray analysis**. (A) Number of probe sets on the *Trichoderma *HDO microarray showing significant expression changes (up- or down-) in *T. harzianum *CECT 2413 in response to the presence of tomato plants (MS-P), chitin (MS-Ch) or glucose (MS-G) in the culture medium in comparison to the basal medium alone (MS). (B) Venn diagrams representing those probe sets that were common and distinct in each culture condition (processed microarray expression data are available in additional file 2).

*Trichoderma *HDO microarrays were hybridized with cDNA obtained from *T. harzianum *CECT 2413 after 9 h of culture in the presence of tomato plants (MS-P medium), chitin (MS-Ch medium), glucose (MS-G medium), or MS basal medium (control condition). From the fluorescence intensities processed as described in Methods, a multi-class SAM test identified a total of 1,617 probe sets (7.0% of the total on the microarray) revealing significant expression changes (FDR = 0.23) between any of the culture conditions under study. Of these probe sets, about 51% had been generated from transcript sequences of *T. harzianum *CECT 2413, and the remaining 49% from transcript sequences of other strains of *Trichoderma*, including 12% of the probe sets from *T. reesei*. The expression data obtained and the identification codes of the corresponding transcript sequences are available as supplementary material in additional file 2. More specifically, we observed that the majority (1,220) of the detected probe sets exhibited a more than two-fold expression change (up- or down-) in one or more culture conditions as compared with the control condition (MS). In particular, 596, 254 and 865 probe sets displayed expression levels at least two-fold higher or lower in MS-P, MS-Ch and MS-G, respectively, than in MS (Figure 2A). In order to determine probe sets specifically related to the presence of tomato plants, we compared those that were common and those that were not common to each culture condition (Figure 2B). Regarding the probe sets reflecting a two-fold higher expression in the presence of tomato plants (MS-P) than in MS, 95 of them (56+11+28) were also found in MS-G and/or MS-Ch, resulting in 162 probe sets (20% of the total up-regulated under the three conditions tested) that were unique to MS-P. Among the probe sets displaying a two-fold lower expression in MS-P than in MS, 110 (37+2+71) were shared with other culture conditions and 229 (35% of the total down-regulated in the three conditions tested) were unique to MS-P.

**Figure 3:**
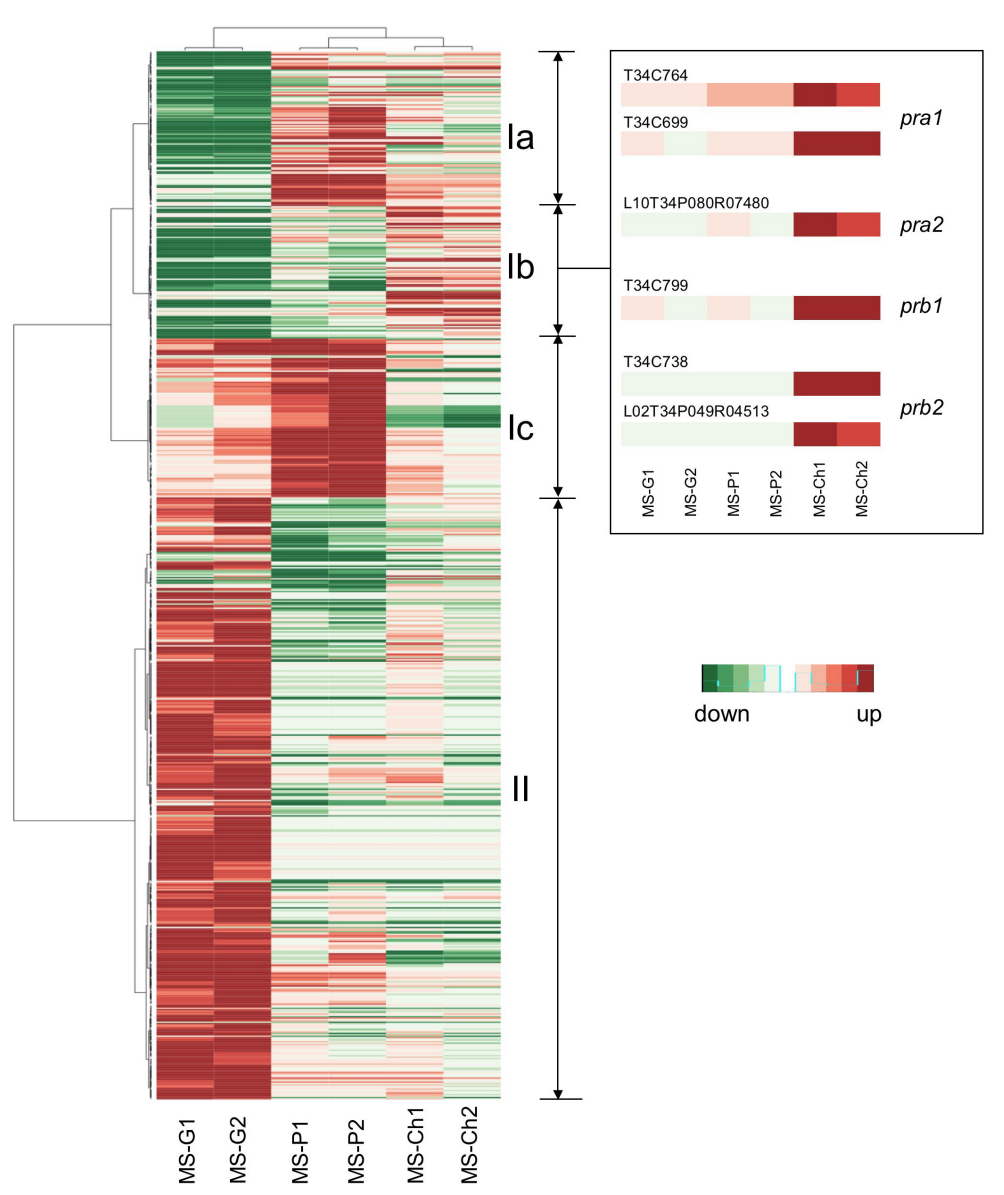
**Heat map representing expression profiles of *T. harzianum *determined by microarray analysis**. A total of 1,220 probe sets showing at least two-fold regulation in response to the presence of tomato plants (MS-P), chitin (MS-Ch) or glucose (MS-G) in the culture medium in comparison to the basal medium alone (MS) were selected for hierarchical clustering. Two biological replicates (1 and 2) from triplicate cultures were used in each experimental condition. Probe sets and samples were ordered using Kendall's *tau *test and the Ward clustering algorithm through the R software. For each row, the mean expression value in the control condition (MS) was calculated and subtracted from the expression value in the rest of conditions. The red and the green colours represent positive and negative expression changes, respectively, *vs*. the control condition. The intensity of the colour is proportional to the magnitude of the differential expression. Detailed expression profiles corresponding to the *pra*1, *pra*2 (former *p7480)*, *prb*1 (former *p10261)*, and *prb*2 (former *p8048*) genes are displayed to the right of the figure (results from different probe sets/ESTs representing the same gene are shown independently).

To gain a general view of the expression data obtained in these microarray experiments, we generated a heat map from the 1,220 probe sets that showed two-fold expression changes in at least one experimental condition *vs*. the MS control condition. Hierarchical clustering was carried out using Kendall's *tau *test and Ward's clustering algorithm since this method resulted in the best resolution of two distinct main clusters, I and II, illustrating different expression patterns (Figure 3). As shown in Figure 3, the two biological replicates of each experimental condition were clustered together. Globally, the majority of the probe sets in the heat map would correspond to genes that are up-regulated by glucose (cluster II, dark red colour) and relatively weakly induced or repressed in the presence of tomato plants and/or chitin (cluster II, light red/green colour). In contrast, probe sets in subclusters Ia and Ib would represent genes that are down-regulated in the presence of glucose but up-regulated in response to tomato plants (mainly in subcluster Ia) or chitin (mainly in cluster Ib). Finally, a subcluster Ic would comprise genes induced by tomato plants and to a certain extent by glucose.

**Figure 4:**
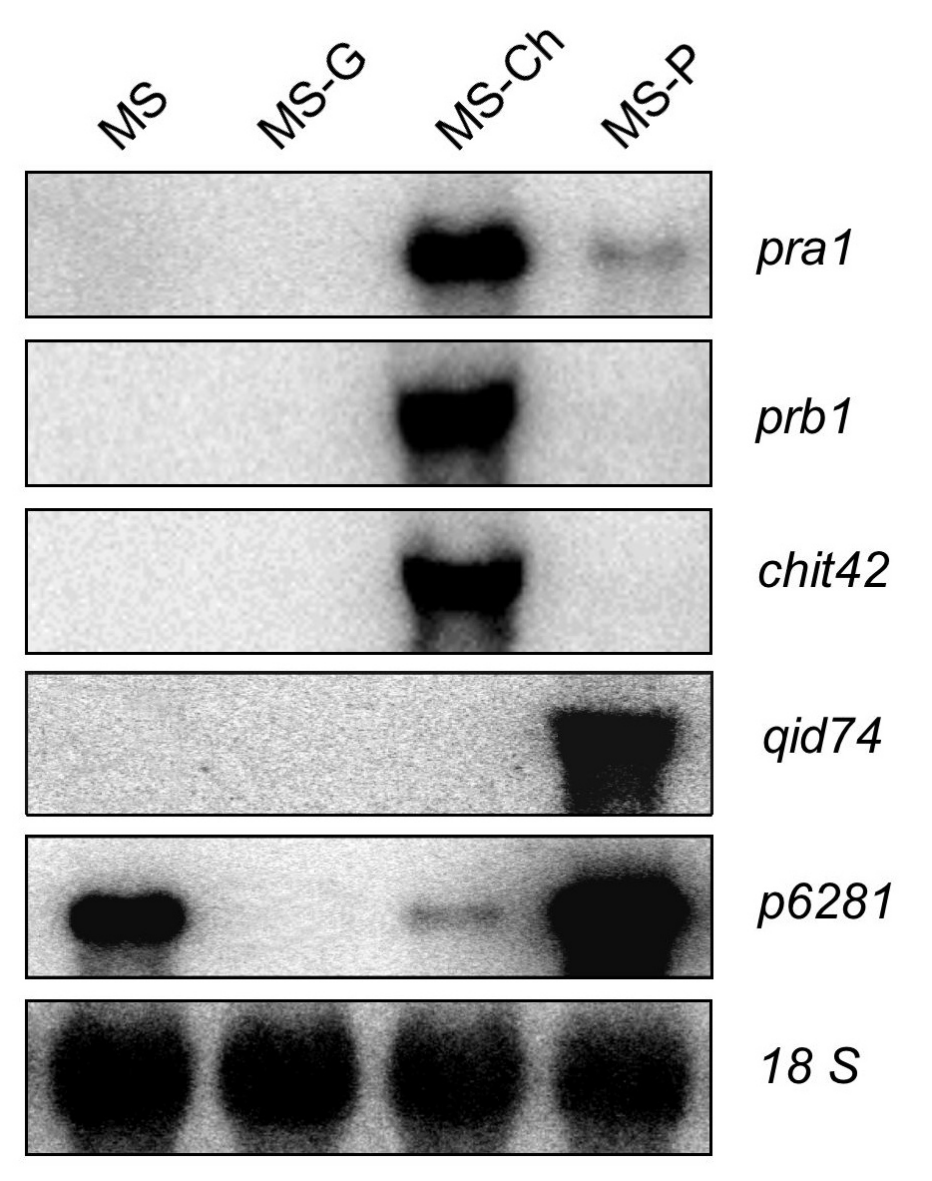
**Expression profiles of five known genes of *T. harzianum *determined by Northern blot hybridization**. The fungus was cultured in MS basal medium alone or in the presence of tomato plants (MS-P), 2% glucose (MS-G), or 1% chitin (MS-Ch), as described in Methods. Fungal 18S rDNA was used as a loading control.

As internal controls of the expression data obtained and the cluster analysis, we searched for probe sets representing genes of *T. harzianum *CECT 2413, such as those coding for trypsins -PRA1 [EMBL: AJ249721] and P7480 (here referred to as PRA2) [EMBL: AM294977]- and subtilisins -P10261 (here referred to as PRB1) [EMBL: AM294980] and P8048 (here referred to as PRB2) [EMBL: AM294978]-, which have been reported to be strongly induced by chitin and repressed by glucose at short-term [26]. As expected, all six probe sets associated with these genes were located in subcluster Ib and yielded expression profiles (Figure 3) consistent with those published previously. Additionally, from the microarray data it was found that these genes exhibited a relatively low level of expression when the fungus was cultured in the presence of tomato plants as compared to that observed when it was cultured in chitin-containing medium. This was also supported by Northern blot analyses carried out for the trypsin PRA1 and subtilisin PRB1 genes. As shown in Figure 4, the transcription of *pra1 *was only weakly triggered in MS-P, whereas the transcript levels of *prb1 *were not even detectable under this condition.

### Identification of *T. harzianum *genes expressed in response to tomato plants

Since we were interested in identifying the genes induced in *T. harzianum *CECT 2413 by the presence of tomato plants, we selected the 257 probe sets affording significant differential expression in MS-P *vs*. MS (fold-change greater than 2.0 and FDR = 0.23; see additional file 3), and the corresponding transcript sequences were annotated according to the GO classification and the hierarchical structure using the Blast2GO suite [27]. GO categories were assigned to 85 of the 257 sequences examined (see additional file 4) whereas another 57 had no results after mapping or annotation processes (many of them were hypothetical proteins), and the remaining 115 sequences did not yield significant hits in the databases. As summarized in additional file 5, the annotated sequences represented a total of 46 different genes. Additionally, three sequences without Blast2GO annotation (T34C26, T34C242 and L10T34P112R10010) but corresponding to three portions of the known protein QID74 [Prot: O74567] of *T. harzianum *CECT 2413 were also included in additional file 5.

Within the genes identified as showing up-regulation in MS-P *vs*. MS, about 45% were genes encoding homologues of proteins involved in metabolic pathways, mainly enzymes for carbohydrate, lipid and amino acid metabolism, but also enzymes for vitamin and cofactor biosynthesis, and energy- and detoxification- related processes. Interestingly, some of these up-regulated genes (encoding O-glycosyl hydrolase family 2, aldose 1-epimerase, dihydroxyacetone kinase, acid sphingomyelin phosphodiesterase, GTP cyclohydrolase I, glutathione-dependent formaldehyde-activating enzyme, plus two hypothetical proteins) were classified according to Blast2GO in the functional category "growth or development of symbiont on or near host surface" since their homologues in *Magnaporte grisea *were differentially expressed during appresorium formation [28]. Proteins related to carbohydrate metabolism included several enzymes of the glycolysis/gluconeogenesis pathways plus a phosphoketolase of the pentose phosphate pathway, and a 1,3-beta-glucan synthase involved in cell wall biosynthesis. The three up-regulated genes with homologues in lipid metabolism corresponded to a phosphatidylserine synthase participating in phospholipid biosynthesis; a dihydroxyacetone kinase involved in glycerolipid metabolism, and an acid sphingomyelin phosphodiesterase, responsible for breaking sphingomyelin down into phosphocholine and ceramide. Proteins related to amino acid metabolism included the enzymes acetylornithine aminotransferase, involved in the urea cycle and the metabolism of amino groups, and 4-hydroxyphenylpyruvate dioxygenase, which catalyzes the third reaction in the catabolism of phenylalanine and tyrosine.

In addition to metabolic genes, we also observed the up-regulated expression in MS-P *vs*. MS of genes involved in signalling, transcription, translation, and post-translational modification and protein folding, including the pH signalling transcription factor Pac1 (PacC) from *T. harzianum *CECT 2413 [EMBL: EF094462]. As shown in additional file 5, genes with homologues in cellular transport and cytoskeleton and cell wall organization were also induced in *T. harzianum *mycelium in the presence of tomato plants.

Interestingly, a homologue of the protein Sm1/Elp1, which is an elicitor of systemic resistance in plants produced by *T. virens/T. atroviride *[29,30], was also found to be induced in *T. harzianum *co-cultured with tomato plants in comparison with the control condition, supporting a role for this gene in the *T. harzianum-*tomato plant interaction. Unexpectedly, some mycoparasitism-associated genes described in the *T. harzianum *CECT 2413 strain, such as those encoding the secreted endochitinase CHIT42 [EMBL: S78423], trypsin-like protease PRA1 [EMBL: AJ249721], aspartic protease P6281 [EMBL: AJ967001] and the cell wall protein QID74 [EMBL: X95671] [31-34], were also significantly up-regulated in the interaction with tomato plants in the absence of phytopathogenic fungi (additional file 5). Northern blot analysis of these genes showed that *p6281 *and *qid74 *were strongly expressed in MS-P, while the transcript levels of *chit42 *and *pra*1 were high in MS-Ch but were scarcely or not detected in MS-P (Figure 4). These results are not surprising, considering that the up-regulated expression of *chit42 *and *pra*1 *vs*. the MS control condition estimated from the microarray hybridizations (additional file 5) resulted from extremely low expression values in this condition (microarray expression data in each culture condition are provided in additional file 2).

### Discussion

This study was undertaken with the dual purpose of constructing an HDO microarray for species of *Trichoderma*, taking advantage of an EST collection previously generated plus the publicly available genome of *T. reesei *[20], and applying this tool for the first time to explore the transcriptional response of a *T. harzianum *biocontrol strain under early (9 h) *Trichoderma*-plant interaction conditions. Other previous approaches at transcriptional level have used macroarray technology to study the interaction of *Trichoderma *spp. with the seedling roots of cacao [13] and tomato [14]. However, the number of cDNA clones represented on these macroarrays -116 in the *Trichoderma *spp.-cacao interaction and 2,496 in the *T. harzianum*-tomato interaction- was much more limited compared with the high capacity, as well as the high reproducibility and sensitivity, of HDO microarrays [35]. Foreman et al. [36] used oligonucleotide microarrays (including 5,131 ESTs) to study the transcriptional regulation of biomass-degrading enzymes from *T. reesei*, a *Trichoderma *sp. of significance in the cellulose industry. In another study, the transcriptome of *T. atroviride *was analyzed using spotted microarrays (1,438 cDNA clones) but again not for the purpose of biocontrol [37].

The analysis reported here is based in a HDO microarray carrying probe sets representative of a total of 23,202 gene transcripts from thirteen *Trichoderma *strains, including 3,826 EST-based transcripts of the *T. harzianum *CECT 2413 biocontrol strain (Figure 1). Despite the redundant nature of EST libraries, a substantial representation of the *T. harzianum *CECT 2413 transcriptome can be expected from the probe sets included on the HDO microarray for this strain, considering that already sequenced *Trichoderma *genomes have been estimated to contain 9,129-11,643 predicted genes [21,22,38]. Moreover, as shown in this work probe sets on the microarray designed from transcripts of *Trichoderma *strains other than *T. harzianum *CECT 2413 were also useful for obtaining information about gene expression in our strain. In particular, we found that nearly half of the probe sets revealing significant expression changes after hybridization with cDNA from *T. harzianum *CECT 2413 (strain T34) derived from other strains or species of *Trichoderma*. The fact that genes known to respond rapidly and sharply to chitin, including those encoding the proteases PRA1, PRA2, PRB1 and PRB2 and the endochitinase CHIT42 [26,39], yielded the expected expression patterns, and that a homologue of the *SM1 *gene with demonstrated expression in the first stages of *T. virens*-root interactions [29] was also detected in our *T. harzianum*-root interaction system, provide a high level of confidence that the microarrays identify differentially expressed genes. We are convinced that at present the *Trichoderma *HDO microarray proposed here offers the opportunity for extensive analyses of gene expression in *Trichoderma *strains whose whole genomes are not scheduled to be sequenced soon, such as those of *T. harzianum*, *T. asperellum *or *T. viride*. An improved microarray may now be possible for *T. virens *and *T. atroviride*, thanks to the release of their genome sequences and the availability of higher-density microarrays that ensure the coverage of complete genomes. For example, gene expression profiling based on entire genome tiling arrays will afford the possibility of monitoring the expression level of whole transcriptomes, avoiding the cloning biases of ESTs and allowing the data arising from different transcript variants that may not have been previously known or predicted to be distinguished. Furthermore, the introduction of new emerging technologies such as massive-scale RNA sequencing will in the near future enable us to overcome some of the limitations inherent to microarray-technology [40].

According to the overall transcriptional profiles, our microarray data showed that changes in gene expression in *T. harzianum *CECT 2413 were more striking (many probe sets displayed the highest or lowest levels of expression) when the fungus was cultured in glucose than with plant roots or with chitin as compared to minimal medium MS, at least at the time examined (9 h; Figure 3). Moreover, the total number of probe sets that exhibited a minimum of two-fold, up- or down-, regulation in glucose was also considerably higher (865) than in the presence of tomato plants (596), and this in turn was higher than in chitin-containing medium (254), with 57% (497), 38% (244), and 18% (45) of the probe sets, respectively, not shared among culture conditions, and hence probably representing genes specifically involved in each particular condition. Globally, the microarray results obtained indicate that *T. harzianum *uses transcriptional controls during its growth in glucose that differ from those occurring in minimal medium (control condition) to a greater extent than they do when the fungus grows on tomato roots and even more when it is grown in a medium containing chitin as the sole carbon source, which could be reasonably correlated with the availability of nutrients to the fungus in each of the culture media. Thus, the larger number of probes sets up-regulated by glucose relative to minimal medium in comparison to other conditions (580 by glucose *vs*. 257 by tomato plants, and 94 by chitin) is consistent with the extensive metabolic activity expected for a filamentous fungus growing in a rich medium with an easily assimilable substrate [41].

The forty-seven distinct genes identified from probe sets whose expression was at least two-fold induced in *T. harzianum *during co-culture with tomato plants (additional file 5) extend the number of previously published induced genes/proteins in *Trichoderma *biocontrol strains during plant colonization to a considerable extent. Nine differential proteins were identified by Marra et al. [15] in *T. atroviride *under *in vitro *interaction conditions with bean plants, using a proteomic approach; using macroarray analysis, Chacón et al. [14] described sixteen induced genes in *T. harzianum *interacting with tomato plant roots; and several more genes have been studied individually, such as those coding for two aspartyl proteases (*pap*A and *pap*B), a hyprophobin (*TasHyd1*) and an expansin-like protein (*TasSwo*) from *T. asperellum*, a mitogen-activated protein kinase (*tmkA/task1*) from *T. virens/T. asperellum*, and a hydrophobin-like protein (*SM1*) belonging to the cerato-platanin family and a non-ribosomal peptide synthetase (*tex*1) from *T. virens *[9-11,29,42,43]. We found that many of the genes induced in *T. harzianum *mycelium in contact with tomato plant roots fell within GO categories related to metabolism, including anabolic and catabolic activities, which indicates an active adaptation of the fungus to the rhizosphere. Six of these genes showed similarities to genes expressed during the formation of infection structures in the phytopathogenic fungus *M. grisea *[28], such as a glycosyl hydrolase belonging to family 2 (with several known hydrolytic activities: beta-galactosidase, beta-mannosidase, and beta-glucuronidase), which was also up-regulated in mycelium of *T. hamatum *and *T. ovalisporum *interacting with cacao seedlings [13]; an aldose 1-epimerase (mutarotase), which is responsible for the anomeric interconversion of D-glucose and other aldoses during normal aldose metabolism [44] and is related to the fungal GAL10 protein, involved in galactose metabolism in *H. jecorina *[45]; a dihydroxyacetone kinase, which uses ATP as a source of high-energy phosphate to produce dihydroxyacetone phosphate, a biochemical compound mainly involved in the glycolytic pathway and lipid biosynthesis; a sphingomyelin phosphodiesterase, a major enzyme for the production of ceramide in response to cellular stresses [46] that also contributes to polarized hyphal growth in *Aspergillus fumigatus *[47], and a gtp cyclohydrolase I, which participates in the production of tetrahydrofolate, in turn involved in nucleic acid and methionine synthesis, and also of tetrahydrobiopterin, a cofactor essential for the synthesis of hydroxy-amino acids, including auxin-related amino acids such as 5-hydroxytryptophan, as well as for the synthesis of nitric oxide (NO). Auxins are important plant regulators involved in many growth and behavioural processes, including those activated by *Trichoderma *spp. [12]. Additionally, NO is a wide-spread signalling molecule related to a number of critical signal transduction pathways in mammals and plants, and it has also been reported to have a regulatory effect in photoconidiation and conidial germination in fungi [48,49]. Another up-regulated gene that suggests that *T. harzianum *could produce NO during the first stages of its interaction with tomato plants is that coding for an acetylornithine aminotransferase, which is a pyridoxal-phosphate-dependent enzyme involved in arginine biosynthesis. L-arginine is important for protein biosynthesis but also participates in the synthesis of NO. In the filamentous fungus Coniothyrium minitans, it has been recently found that arginine is essential for conidiation, possibly through a NO-mediated process [50].

Another ten identified genes induced in *T. harzianum *by the presence of tomato plants also pointed to the active growth and development of the fungus, among them, those encoding homologues of two D-lactate dehydrogenases, which modulate the flow of pyruvate when glucose is required for cell growth or hyphal development [51]; a glucan synthase, which is a key enzyme for fungal cell wall biosynthesis [52] and whose up-regulation is correlated with the previous proteomic study performed by Marra et al. [15] showing increased expression of a cell wall synthesis-associated chitin synthase in *T. atroviride *during interaction with bean leaves; a phosphatidylserine synthase, which is known to participate in cell-membrane building through the CDP-diacylglycerol metabolic process [53]; a 4-hydroxyphenylpiruvate dioxigenase, which has been found to be directly involved in the growth and differentiation of the pathogenic phase of the fungus Paracoccidioides brasiliensis [54]; a formyltetrahydrofolate deformylase, which participates in small-molecule metabolism and the synthesis of DNA and has been described to be related to early stages of microbial symbiotic relations to plants [55]; a pentatricopeptide repeat (PPR) protein belonging to the PPR protein family that includes proteins involved in RNA post-transcriptional processing and development [56]; and a class II hydrophobin from *T. virens *[EMBL: ABS59373] [57]. *Trichoderma *hydrophobins are known to play major roles in hyphal development and conidiation [58-60], and it has also been reported recently that the hydrophobin TasHyd1 from *T. asperellum *participates in cucumber root colonization [9] and that a hydrophobin of *T. atroviride *was induced during interaction with bean roots [15].

Another gene related to adhesion to hydrophobic surfaces with up-regulated expression in *T. harzianum *in response to growth on tomato plant roots was *qid*74. This gene, which encodes a cell wall protein described in *T. harzianum *CECT 2413 [34], has been proposed to participate in cell adherence and cell wall protection against toxins produced by fungal hosts during mycoparasitic interactions [61], and our expression results support a role for this protein in the *Trichoderma*-plant interaction. Apart from *qid74*, the significant up-regulation detected by microarrays of some other known genes of *T. harzianum *CECT 2413, such as those encoding secreted enzymes associated with the breakdown of fungal cell wall components during *Trichoderma*-fungal host interactions (endochitinase CHIT42 and proteases PRA1 and P6281) and the transcription factor Pac1 (which was been described to regulate some of these micoparasitic enzymes [62]), indicates that they could also participate during early stages of the *Trichoderma*-plant interaction. These data match the hypothesis suggested by Woo et al. [5] that among the *Trichoderma *elicitors that activate plant defence responses are some of the proteins, such as chitinases and glucanases, that the fungus uses for its mycoparasitic activities.

The increased expression in *T. harzianum*-tomato plant co-cultures of a nitropropane dioxygenase gene, which has been related to fungal defence against toxic nitroalkanes produced by plants [63], suggests that *T. harzianum *is able to protect itself from harmful plant substances. Some other up-regulated genes that may also be related to detoxification processes were those encoding a dimethylaniline monooxygenase, which is a broad spectrum flavoprotein that accepts diverse substrates, including plant alkaloids [64]; a RTA-1 domain protein belonging to the RTA1 family, which is comprised of fungal proteins involved in resistance to toxic substances [65]; and a glutathione-dependent formaldehyde-activating enzyme, which could be involved in the consumption of the cytotoxic formaldehyde resulting from many demethylation reactions [66].

Finally, it is worth noting that those non-identified transcripts that were detected in this study as up-regulated in *T. harzianum *by the presence of tomato plants (non-annotated sequences from additional file 3) are also an additional resource for future research on *Trichoderma*-plant interactions, especially those that did not respond significantly to other culture conditions assessed.

## Conclusion

The *Trichoderma *HDO microarray presented here has enabled us to define a gene set probably involved in the transcriptional response of the fungus *T. harzianum *CECT 2413 within the first hours of contact with tomato plant roots. Many of the genes identified had not been previously related to *Trichoderma*-plant interactions, including those respsonsible for the possible biosynthesis of nitric oxide, xenobiotic detoxification, micoparasitic activities, mycelium development, or those related to the formation of infection structures in plant tissues, which can provide new insight into the mechanisms and roles of this fungus in the *Trichoderma-*plant interaction. The effectiveness of the *Trichoderma *HDO microarray in the detection of different gene responses in *T. harzianum *under different growth conditions strongly indicates that this tool should be useful for further assays addressing different stages of plant colonization, as well as for expression studies in other *Trichoderma *spp. represented on it.

## Methods

### Fungal and plant growth conditions

*Trichoderma harzianum *CECT 2413 (Spanish Type Culture Collection, Valencia, Spain) was grown on potato dextrose agar (Sigma, St. Louis, Mo, USA) plates in the dark at 28°C for 10 days. Spores were collected and used as inoculum (10^7 ^spores as counted with a hemocytometer) for fungal pre-cultures in 250-ml Erlenmeyer flasks containing 100 ml of liquid minimal medium [67] supplemented with 2% glucose as carbon source. Flasks were then maintained at 28°C and 150 rpm for 48 h. After this time, fungal biomass was harvested by filtration, washed twice with sterile distilled water, and immediately transferred to the definitive cultures (see below).

Tomato seeds (*Solanum lycopersicum*, formerly *Lycopersicon esculentum *Mill. var. Manitu) from Ramiro Arnedo S.A. (Calahorra, La Rioja, Spain) were surface-sterilized by vigorous sequential shaking in 70% ethanol and 2% hypochlorite solution, for 5 min each, and then thoroughly washed with sterile distilled water and air-dried on a sterile gauze sheet. Seeds were germinated in multi-cell growing trays containing sterile soil substrate covered with vermiculite in a controlled environment chamber with 75% humidity and a photoperiod of 16 h light at 23°C. Plants were then allowed to grow under these conditions for twelve weeks.

For *Trichoderma-*plant interactions in hydroponic cultures, twelve-week-old tomato plants were collected and their roots were thoroughly washed in sterile distilled water, and surface sterilized by dipping sequentially in 70% ethanol, 2% hypochlorite solution, and sterile distilled water. Then, each tomato plant was submerged up to the stem in a 250-ml Erlenmeyer flask filled with 100 ml of liquid Murashige and Skoog (MS) basal medium (Duchefa, Haarlem, The Netherlands) (MS-P medium). MS is a commonly used medium for plant tissue cultures but it has been also used to analyze *Trichoderma *secreted proteins in hydroponic systems [8,14]. Immediately, *T. harzianum *mycelia obtained as described above were also transferred to the MS-P medium under aseptic conditions. Fungal cultures in MS medium without the presence of tomato plants were used as controls.

*T. harzianum *cultures in rich medium (MS supplemented with 2% glucose: MS-G medium) and in the presence of chitin [MS containing 1% chitin (Sigma, St. Louis, Mo, USA): MS-Ch medium] were also included in the study for comparative purposes.

All cultures were maintained at 28°C and 90 rpm for 9 h. After this time, *Trichoderma *mycelia were harvested by filtration (the mycelium on the plant roots was recovered with a direct water jet, avoiding excessive manipulation). Mycelia were washed twice with sterile distilled water, frozen in liquid nitrogen, lyophilized, and kept at -80°C until RNA extraction.

### Microarray design and construction

A self-designed Trichoderma high-density oligonucleotide (HDO) microarray was used in this study. A collection of 14,237 transcript sequences obtained for the "TrichoEST project" from ESTs (11,376 singlets and 2,861 contigs provided in additional files 6 and 7, respectively) of twelve strains of eight different Trichoderma spp. [CECT: T. harzianum T34 (CECT 2413); NewBiotechnic S.A. (NBT, Seville, Spain): T. longibrachiatum T52 (NBT52); T. virens T59 (NBT59), T. viride T78 (NBT78); American type Culture Collection (ATCC, Rockville, USA): T. atroviride TP1 (ATCC 74058), T. harzianum T22 (ATCC 20847); Centraalbureau voor Schimmelcultures (CBS, Baarn, The Netherlands): T. stromaticum TST (CBS 100875); International Mycological Institute (IMI, Egham, UK): T. atroviride T11 (IMI 352941); T. asperellum T53 (IMI 20268); BioCentrum-DTU Culture Collection of Fungi (IBT, Lyngby, Denmark): T. harzianum T3K (IBT 9385); T. aggressivum TH2 (IBT 9394); University Federico II of Naples (UNINA, Portici, Italy): T. harzianum TA6 (UNINA 96)], plus 9,129 transcript sequences predicted from the T. reesei QM 6a genome [38] were used as source sequences to generate probes for the Trichoderma HDO microarray.

First, unique sequences were obtained from the whole TrichoEST database by combining ESTs from all twelve Trichoderma strains indicated above in order to minimize redundancy due to transcripts common to different strains. CAP3 assembly [68] of the combined ESTs resulted in 3,152 contigs and 9,510 singlets, totalling 12,662 unique sequences. The probe selection process was then carried out by 'in-house' bioinformatics programs, executing the following steps: (1) An initial pool of all possible probes was obtained by sliding a 25-bp window with a step-size of 1-bp over each source sequence (12,662 + 9,129), resulting in a total of 18,881,401 different probes. (2) Then, the probes were matched against the total of source sequences and additionally against the full-length genome of T. reesei to evaluate their uniqueness by simple frequency counting. The probes that matched more than one transcript of T. reesei or more than fifty transcripts of Trichoderma spp. or that occurred more than once in the complete T. reesei genome were discarded by the probe selection algorithm. A frequency cut-off of 50 was set with respect to the Trichoderma EST-based database with the aim of covering redundant sequences that remained erroneously unassembled into contigs, for example, due to residual vector contaminations. (3) The resulting probe list (18,870,469 probes) was further narrowed by applying different probe quality filters: self-complementarity; a GC-content between 40-60%; a content of any single nucleotide less than 40% of the probe length; fewer than five consecutive nucleotide repetitions. (4) Finally, a probe prioritization process was carried out to adjust the total number of probes that passed the previous criteria (6,060,523 probes) to the microarray capacity (385,000 probes). To accomplish this, probes were first mapped to both Trichoderma spp. and T. reesei transcript sequence collections and were then evenly spaced over each sequence with a fixed minimum number of 10 probes per sequence (or 10 probes within a probe set), except for those with less than 10 probes passing the previous filters. Since a random priming strategy was to be used for cDNA sample preparation, probes were distributed uniformly along each whole transcript sequence.

The final probe list was submitted to Roche-NimbleGen, Inc. (Madison, WI, USA) for quality control and subsequent probe array layout. Additional probes were also included on the microarray by Roche-NimbleGen, Inc. for quality control of the hybridization process. Microarray manufacture was then carried out using maskless, digital micromirror technology [69].

### Sample preparation for microarray hybridization

*T. harzianum *CECT 2413 freeze-dried mycelia were ground in liquid nitrogen using a mortar and pestle, and total RNA was extracted using TRIzol^® ^reagent (Invitrogen Life Technologies, Carlsbad, CA, USA), according to the manufacturer's instructions. The RNA quality and quantity were determined spectrophotometrically and the RNA integrity was confirmed by agarose gel electrophoresis. For each experimental condition, an equal amount of total RNA (200 μg) from three independent replicates of mycelium was mixed. mRNA was then purified using Dynabeads (Dynal^®^, Oslo, Norway) twice consecutively to avoid rRNA contamination. Then, cDNA synthesis was performed from 5 μg mRNA using the Just cDNA™ Double-Stranded cDNA Synthesis Kit (Stratagene, La Jolla, CA, USA), according to the manufacturer's instructions. A random priming strategy was followed in order to obtain cDNAs with more 5' information. The cDNAs were finally submitted to NimbleGen Systems Inc. for labelling with Cy3 dye-labelled 9 mer random primers and subsequent hybridization using a MAUI (Micro Array User Interface) Hybridization System (.BioMicro^® ^Systems, Salt Lake City, UT, USA). Hybridizations were carried out in duplicate with cDNA obtained from independent experiments.

### Microarray data analysis

Microarray scanning and data acquisition were performed by NimbleGen Systems Inc. using an Axon GenePix 4000B scanner with associated NimbleScan 2.3 software. Then, the images and the raw probe intensity values obtained from the eight microarrays were examined, processed, and analysed at our lab. The raw data were deposited in the GEO database [70] with series accession number GSE13776. Visual inspection of the scanned images failed to reveal obvious scratches or spatial variations across each microarray. Similarly, the distributions of the raw probe intensities were generated for all microarrays, and no apparent deviances were observed. Data were subsequently processed for background adjustment, normalization and summarization. Briefly, a Robust Multichip Average (RMA) convolution model was applied for background correction, and the corrected probe intensities were then normalized using a quantile-based normalization procedure as performed by Irizarry et al. [71]. Following this, the normalized values for each probe obtained from the eight microarrays were scaled in the 0-1 range to compensate for sequence-specific sensitivity. Finally, the processed data for the different probes within a probe set were summed to produce an expression measure.

To identify probe sets showing a significant difference in expression level in at least one of the culture conditions considered (fungus grown in MS-P, MS-Ch, MS-G and MS) compared to one another, a multi-class Significance Analysis of Microarray (SAM) test [72] was carried out on the expression values using a False Discovery Rate (FDR) of 0.23. The analysis was performed using the siggenes package [73] through the R software environment for statistical computing and graphics [74].

Transcripts showing significantly up-regulated expression were annotated using Gene Ontology (GO) terms and hierarchical structure http://www.geneontology.org. The Blast2GO program [27], which assigns the GO terms based on the BLAST definitions, was applied with an E-value < 10^-5 ^level.

### Northern blot analyses

Northern blots were obtained using total RNA extracted from *T. harzianum *CECT 2413 freeze-dried mycelia collected as described above. RNA separation (30 μg), blotting and hybridization were carried out using standard techniques. Specific DNA probes of each gene were produced by PCR of the corresponding cDNA clone from our *Trichoderma *cDNA clone collection, using the primers T3 and T7 from the Uni-Zap XR vector (Stratagene). The DNA probes were P^32^-labelled using Ready to go DNA labelling beads (Amersham Biosciences, Freiburg, Germany) and radioactive signals were visualized with a PhosphorImager System (Bio-Rad, Hercules, CA, USA), using QuantityOne software.

## Authors' contributions

IS performed the experiments and helped with the interpretation of the data. ADL designed and developed the probe selection process and performed the bioinformatics and statistical analyses of microarray data. JAV performed the sequence annotation and revised the manuscript. EMV supervised the study and helped in writing the discussion of the manuscript. MBS designed and coordinated the study, participated in the experiments, the microarray data analysis and the annotation process, and wrote the manuscript. All authors read and approved the final manuscript.

## Supplementary Material

Additional file 1 **Table S1**. Identification codes of the *Trichoderma *sp. (EST-derived) and *T. reesei *(genome-derived) transcripts that were excluded from the *Trichoderma *HDO microarray. Click here for file

Additional file 2 **Table S2**. List of 1,617 *Trichoderma *transcripts whose probe sets afforded a significant difference in expression levels (FDR = 0.23) in microarray experiments in at least one of the culture conditions considered: *T. harzianum *CECT 2413 grown for 9 hours in MS medium in the presence of tomato plants (MS-P), chitin (MS-Ch), glucose (MS-G), or MS basal medium alone. Click here for file

Additional file 3 T**able S3**. List of 257 selected *Trichoderma *transcripts whose probe sets afforded significant up-regulation (fold-change higher that 2.0 and FDR = 0.23) in microarray experiments after hybridization with cDNA from *T. harzianum *CECT 2413 grown for 9 hours in MS medium in the presence of tomato plants (MS-P) in comparison with the control condition in MS medium alone. Expression values of these probe sets obtained from the fungus grown in chitin- (MS-Ch) and glucose- (MS-G) containing MS media are also shown. Click here for file

Additional file 4 **Table S4**. List of 85 annotated transcript sequences of *Trichoderma *spp. whose probe sets showed significant up-regulation (fold-change greater than 2.0 and FDR = 0.23) in microarray experiments after hybridization with cDNA from *T. harzianum *CECT 2413 grown for 9 hours in interaction with tomato plants in MS medium compared with the control condition in MS medium alone. Biological processes (P), molecular functions (F) and cellular components (C) are based on Gene Ontology (GO) categories inferred from electronic annotation using the Blast2GO suite based on BLAST definitions. Click here for file

Additional file 5 **Table S5**. Genes induced in *T. harzianum *in contact with tomato plant roots. Click here for file

Additional file 6 **Table S6**. EMBL database accession numbers of the *Trichoderma *ESTs used in this study. Click here for file

Additional file 7 T**able S7**. *Trichoderma *ESTs that cluster in each contig. Click here for file
