# Supplementary material for: Gene expression analysis of the biocontrol fungus Trichoderma harzianum in the presence of tomato plants, chitin, or glucose using a high-density oligonucleotide microarray
Source: BMC Microbiol. 2009 Oct 13;9:217. doi: 10.1186/1471-2180-9-217 (PMC2768740; doi:10.1186/1471-2180-9-217)
Supplement: Additional file 4 — Table S4. List of 85 annotated transcript sequences of Trichoderma spp. whose probe sets showed significant up-regulation (fold-change greater than 2.0 and FDR = 0.23) in microarray experiments after hybridization with cDNA from T. harzianum CECT 2413 grown for 9 hours in interaction with tomato plants in MS medium compared with the control condition in MS medium alone. Biological processes (P), molecular functions (F) and cellular components (C) are based on Gene Ontology (GO) categories inferred from electronic annotation using the Blast2GO suite based on BLAST definitions. [file 1471-2180-9-217-S4.PDF]

Table S4. List of 85 annotated transcript sequences of Trichoderma spp. whose probe sets showed significant up-regulation (fold-change greater than 2.0 and FDR = 0.23) in microarray experiments after hybridization with cDNA from T. harzianum CECT 2413 grown for 9 hours in interaction with tomato plants in MS medium compared with the control condition in MS medium alone. Biological processes (P), molecular functions (F) and cellular components (C) are based on Gene Ontology (GO) categories inferred from electronic annotation using the Blast2GO suite based on BLAST definitions.

SeqName	Hit description	Length	Hits	min. eValue	sim mean	GOs	GO IDs	GO description
L50TH2P008R00685	protein	557	10	1.0E-1.23461E-30	52.0%	1	P:GO:0044408	P:growth or development of symbiont on or near host surface
L50TH2P011R01007	mitochondrial atpase	574	12	1.0E-1.69034E-17	77.5%	3	F:GO:0004857<br>P:GO:0045980<br>C:GO:0005739	F:enzyme inhibitor activity<br>P:negative regulation of nucleotide metabolic process<br>C:mitochondrion
L50TH2P014R01277	protein	323	8	1.0E-1.5141E-8	66.5%	1	C:GO:0005739	C:mitochondrion
L50TH2P015R01434	protein	443	8	1.0E-1.42712E-11	61.88%	1	C:GO:0005739	C:mitochondrion
L50TH2P017R01593	protein	462	15	1.0E-2.51705E-16	74.0%	1	C:GO:0005739	C:mitochondrion
L50TH2P017R01594	protein	461	16	1.0E-2.14013E-15	74.63%	1	C:GO:0005739	C:mitochondrion
L50TH2P020R01889	dynein heavy chain	646	20	1.0E-2.16184E-72	81.35%	7	C:GO:0005737<br>P:GO:0007018<br>C:GO:0030286<br>F:GO:0016887<br>F:GO:0005524<br>F:GO:0003777<br>C:GO:0005874	C:cytoplasm<br>P:microtubule-based movement<br>C:dynein complex F:ATPase activity F:ATP binding<br>F:microtubule motor activity<br>C:microtubule
TH2C17	protein	459	16	1.0E-8.13249E-15	72.69%	1	C:GO:0005739	C:mitochondrion
TH2C110	protein	424	8	1.0E-6.79871E-9	65.0%	1	C:GO:0005739	C:mitochondrion
L51TP1P002R00166	60s ribosomal protein l40	511	20	1.0E-3.85831E-16	93.2%	9	F:GO:0031386<br>P:GO:0042254<br>F:GO:0003735<br>P:GO:0016567<br>C:GO:0022625<br>C:GO:0005634<br>P:GO:0006412<br>C:GO:0005739	F:protein tag<br>P:ribosome biogenesis and assembly<br>F:structural constituent of ribosome<br>P:protein ubiquitination<br>C:cytosolic large ribosomal subunit

							P:GO:0006281	C:nucleus<br>P:translation<br>C:mitochondrion<br>P:DNA repair
L51TP1P003R00220	gtp cyclohydrolase i	572	20	1.0E-2.89653E-62	85.8%	5	C:GO:0005737<br>P:GO:0006729<br>F:GO:0003934<br>P:GO:0044408<br>F:GO:0008270	C:cytoplasm<br>P:tetrahydrobiopterin biosynthetic process<br>F:GTP cyclohydrolase I activity<br>P:growth or development of symbiont on or near host surface<br>F:zinc ion binding
L51TP1P003R00268	protein	431	16	1.0E-1.95714E-16	71.25%	1	C:GO:0005739	C:mitochondrion
L51TP1P005R00418	protein	421	8	1.0E-6.82695E-9	64.38%	1	C:GO:0005739	C:mitochondrion
L51TP1P006R00499	protein	548	12	1.0E-2.99381E-18	73.58%	4	P:GO:0042110<br>C:GO:0005737<br>F:GO:0005488<br>P:GO:0007249	P:T cell activation<br>C:cytoplasm<br>F:binding<br>P:I-kappaB kinase/NF-kappaB cascade
L51TP1P019R01734	prefoldin subunit	549	20	1.0E-1.31265E-45	81.55%	3	P:GO:0006457<br>C:GO:0016272<br>F:GO:0051082	P:protein folding<br>C:prefoldin complex<br>F:unfolded protein binding
L54TP1P031R02884	and tpr domain protein	506	20	1.0E-1.16988E-41	78.2%	2	P:GO:0043581<br>F:GO:0031072	P:mycelium development<br>F:heat shock protein binding
TP1C33	outer membrane protein	550	20	1.0E-1.11639E-28	79.9%	2	P:GO:0015031<br>C:GO:0005741	P:protein transport<br>C:mitochondrial outer membrane
TP1C190	protein	396	16	1.0E-2.58921E-16	73.44%	1	C:GO:0005739	C:mitochondrion
TP1C204	protein	518	8	1.0E-4.31546E-10	63.5%	1	C:GO:0005739	C:mitochondrion
TP1C260	protein	414	16	1.0E-1.25401E-15	73.19%	1	C:GO:0005739	C:mitochondrion
L52T3KP009R00825	---NA---	452	7	1.0E-1.40531E-14	83.0%	3	F:GO:0004857<br>P:GO:0045980<br>C:GO:0005739	F:enzyme inhibitor activity<br>P:negative regulation of nucleotide metabolic process<br>C:mitochondrion
L52T3KP015R01357	d-lactate dehydrogenase	478	20	1.0E-7.3557E-32	73.05%	4	F:GO:0051287<br>P:GO:0043581<br>F:GO:0016616	F:NAD binding<br>P:mycelium development<br>F:oxidoreductase activity,

							P:GO:0008152	acting on the CH-OH group of donors, NAD or NADP as acceptor<br>P:metabolic process
L52T3KP019R01759	dihydroxyacetone kinase	551	20	1.0E-3.96144E-34	78.3%	4	P:GO:0006071<br>F:GO:0005524<br>F:GO:0004371<br>P:GO:0044408	P:glycerol metabolic process<br>F:ATP binding<br>F:glycerone kinase activity<br>P:growth or development of symbiont on or near host surface
T3KC61	---NA---	486	7	1.0E-1.41762E-14	83.0%	3	F:GO:0004857<br>P:GO:0045980<br>C:GO:0005739	F:enzyme inhibitor activity<br>P:negative regulation of nucleotide metabolic process<br>C:mitochondrion
T3KC128	protein	436	14	1.0E-5.84342E-13	72.78%	1	C:GO:0005739	C:mitochondrion
L20T59P001R00153	hydrophobin	623	20	1.0E-8.41289E-27	63.3%	3	P:GO:0045230<br>P:GO:0030448<br>C:GO:0030446	P:capsule organization and biogenesis<br>P:hyphal growth<br>C:hyphal cell wall
T59C72	---NA---	571	6	1.0E-1.31841E-14	85.33%	3	F:GO:0004857<br>P:GO:0045980<br>C:GO:0005739	F:enzyme inhibitor activity<br>P:negative regulation of nucleotide metabolic process<br>C:mitochondrion
L55TSTP004R00316	ubiquitin conjugating	580	20	1.0E-1.77015E-53	69.5%	3	P:GO:0051246<br>F:GO:0019787<br>P:GO:0043687	P:regulation of protein metabolic process<br>F:small conjugating protein ligase activity<br>P:post-translational protein modification
L55TSTP005R00477	tar1_yeastame: full=protein tar1ame: full=transcript antisense to ribosomal rna protein 1	382	3	1.0E-2.16854E-7	72.66%	1	C:GO:0005739	C:mitochondrion
L57TSTP019R01790	protein	501	13	1.0E-5.17985E-10	69.30%	1	C:GO:0005739	C:mitochondrion
L57TSTP020R01832	protein	462	15	1.0E-1.02191E-17	73.46%	1	C:GO:0005739	C:mitochondrion
L57TSTP020R01889	protein	543	8	1.0E-4.25572E-9	61.625%	1	C:GO:0005739	C:mitochondrion
TSTC12	protein	488	8	1.0E-1.01593E-12	61.375%	1	C:GO:0005739	C:mitochondrion
TSTC55	protein	452	8	1.0E-7.97983E-10	63.875%	1	C:GO:0005739	C:mitochondrion

TSTC161	protein	307	14	1.0E-8.43237E-12	72.85%	1	C:GO:0005739	C:mitochondrion
TSTC171	tar1_yeastame: full=protein tar1ame: full=transcript antisense to ribosomal rna protein 1	519	7	1.0E-1.08555E-8	65.14%	1	C:GO:0005739	C:mitochondrion
L21T78P012R01118	---NA---	586	6	1.0E-1.42305E-14	85.16%	3	F:GO:0004857<br>P:GO:0045980<br>C:GO:0005739	F:enzyme inhibitor activity<br>P:negative regulation of nucleotide metabolic process<br>C:mitochondrion
L21T78P017R01602	---NA---	586	7	1.0E-2.42735E-14	83.14%	3	F:GO:0004857<br>P:GO:0045980<br>C:GO:0005739	F:enzyme inhibitor activity<br>P:negative regulation of nucleotide metabolic process<br>C:mitochondrion
L92S34P002R00160	phosphatidylserine synthase	546	20	1.0E-6.03232E-19	75.05%	4	P:GO:0043581<br>F:GO:0016780<br>C:GO:0044464<br>P:GO:0008654	P:mycelium development<br>F:phosphotransferase activity, for other substituted phosphate groups<br>C:cell part<br>P:phospholipid biosynthetic process
L92S34P005R00458	glucan synthase	570	20	1.0E-3.46951E-15	76.0%	1	C:GO:0016020	C:membrane
T34C294	aspartic peptidase a1	671	20	1.0E-3.90165E-100	63.95%	2	P:GO:0006508<br>F:GO:0004194	P:proteolysis<br>F:pepsin A activity
T34C720	---NA---	761	6	1.0E-4.22052E-14	84.83%	3	F:GO:0004857<br>P:GO:0045980<br>C:GO:0005739	F:enzyme inhibitor activity<br>P:negative regulation of nucleotide metabolic process<br>C:mitochondrion
T34C721	glycosyl hydrolase family	779	20	1.0E-5.27866E-100	70.65%	4	F:GO:0004553<br>P:GO:0005975<br>P:GO:0044408<br>F:GO:0043169	F:hydrolase activity, hydrolyzing O-glycosyl compounds<br>P:carbohydrate metabolic process<br>P:growth or development of symbiont on or near host surface<br>F:cation binding
T34C798	glucan synthase	751	20	1.0E-2.77137E-42	80.7%	3	C:GO:0000148<br>F:GO:0003843	C:1,3-beta-glucan synthase complex

							P:GO:0006075	F:1,3-beta-glucan synthase activity<br>P:1,3-beta-glucan biosynthetic process
L07T11P033R03099	protein	643	20	1.0E-7.08976E-48	77.25%	1	C:GO:0005739	C:mitochondrion
L02T34P003R00286	---NA---	518	7	1.0E-3.65325E-9	87.57%	3	F:GO:0004857<br>P:GO:0045980<br>C:GO:0005739	F:enzyme inhibitor activity<br>P:negative regulation of nucleotide metabolic process<br>C:mitochondrion
L02T34P018R01663	ccr4_neucrame: full=glucose-repressible alcohol dehydrogenase transcriptional effectorame: full=carbon catabolite repressor protein 4ame: full=cytoplasmic deadenylase	584	20	1.0E-4.49697E-35	77.45%	8	C:GO:0005737<br>F:GO:0004535<br>C:GO:0042025<br>F:GO:0003723<br>F:GO:0005515<br>F:GO:0000287<br>C:GO:0005634<br>P:GO:0006355	C:cytoplasm<br>F:poly(A)-specific ribonuclease activity<br>C:host cell nucleus<br>F:RNA binding<br>F:protein binding<br>F:magnesium ion binding<br>C:nucleus<br>P:regulation of transcription, DNA-dependent
L03T34P047R04348	epl1 protein	687	20	1.0E-3.04634E-18	79.55%	2	P:GO:0052051<br>P:GO:0009405	P:interaction with host via protein secreted by type II secretion system<br>P:pathogenesis
L03T34P059R05502	pentatricopeptide repeat protein	625	20	1.0E-6.86647E-21	81.55%	4	P:GO:0008615<br>F:GO:0004733<br>P:GO:0043581<br>F:GO:0010181	P:pyridoxine biosynthetic process<br>F:pyridoxamine-phosphate oxidase activity<br>P:mycelium development<br>F:FMN binding
L03T34P070R06566	---NA---	572	3	1.0E-2.84483E-9	84.66%	3	F:GO:0004857<br>P:GO:0045980<br>C:GO:0005739	F:enzyme inhibitor activity<br>P:negative regulation of nucleotide metabolic process<br>C:mitochondrion
L11T34P092R08690	aldose 1-	364	20	1.0E-2.44443E-19	77.75%	4	P:GO:0005975<br>F:GO:0030246<br>F:GO:0016853<br>P:GO:0044408	P:carbohydrate metabolic process<br>F:carbohydrate binding<br>F:isomerase activity<br>P:growth or development of symbiont on or near host

								surface
L11T34P099R09379	aldose 1-	425	20	1.0E-2.49075E-19	77.75%	4	P:GO:0005975<br>F:GO:0030246<br>F:GO:0016853<br>P:GO:0044408	P:carbohydrate metabolic process<br>F:carbohydrate binding<br>F:isomerase activity<br>P:growth or development of symbiont on or near host surface
jgi|Trire2|123468|estExt_fgenesh5_pg.C_220056	oxidoreductase 2-nitropropane dioxygenase	1301	20	1.0E-1.04158E-103	69.8%	1	F:GO:0016491	F:oxidoreductase activity
TSTC58	protein	573	10	1.0E-1.63723E-12	60.9%	1	C:GO:0005739	C:mitochondrion
TSTC15	protein	555	17	1.0E-1.24035E-14	68.11%	1	C:GO:0005739	C:mitochondrion
TSTC134	glutathione-dependent formaldehyde-gfa	513	20	1.0E-2.81995E-22	50.6%	1	P:GO:0044408	P:growth or development of symbiont on or near host surface<br>F:carbon-sulfur lyase activity
TSTC120	ribosomal s30 ubiquitin fusion	384	20	1.0E-2.8932E-16	87.55%	3	C:GO:0005840<br>F:GO:0003735<br>P:GO:0006412	C:ribosome<br>F:structural constituent of ribosome<br>P:translation
TP1C80	protein	588	9	1.0E-1.91451E-11	62.11%	1	C:GO:0005739	C:mitochondrion
TH2C96	protein	536	9	1.0E-1.47749E-11	62.11%	1	C:GO:0005739	C:mitochondrion
T3KC86	protein	583	9	1.0E-1.11097E-11	61.55%	1	C:GO:0005739	C:mitochondrion
T34C611	acetylornithine aminotransferase	528	20	1.0E-1.40016E-30	72.95%	4	P:GO:0006525<br>F:GO:0008483<br>P:GO:0008652<br>C:GO:0005739	P:arginine metabolic process<br>F:transaminase activity<br>P:amino acid biosynthetic process<br>C:mitochondrion
T34C557	translationally controlled tumorvariant i	661	20	1.0E-1.16558E-64	79.35%	6	C:GO:0005840<br>P:GO:0006979<br>P:GO:0006412<br>C:GO:0005829<br>C:GO:0005739<br>C:GO:0005874	C:ribosome<br>P:response to oxidative stress<br>P:translation<br>C:cytosol<br>C:mitochondrion<br>C:microtubule
T34C109	profilin-like protein	659	20	1.0E-4.42136E-32	58.7%	7	P:GO:0007114<br>P:GO:0000282	P:cell budding<br>P:cellular bud site selection

							P:GO:0030036<br>F:GO:0003779<br>C:GO:0015629<br>P:GO:0046907<br>C:GO:0005737	P:actin cytoskeleton organization and biogenesis<br>F:actin binding<br>C:actin cytoskeleton<br>P:intracellular transport<br>C:cytoplasm
L53TP1P028R02620	60s ribosomal protein l44	443	20	1.0E-3.61236E-31	80.7%	5	P:GO:0046898<br>F:GO:0003735<br>C:GO:0022625<br>P:GO:0006412<br>P:GO:0046677	P:response to cycloheximide<br>F:structural constituent of ribosome<br>C:cytosolic large ribosomal subunit<br>P:translation<br>P:response to antibiotic
L52T3KP010R00917	lyr family protein	484	20	1.0E-3.72682E-44	80.8%	2	C:GO:0005743<br>F:GO:0016651	C:mitochondrial inner membrane<br>F:oxidoreductase activity, acting on NADH or NADPH
L52T3KP007R00584	---NA---	585	9	1.0E-2.96036E-20	60.22%	1	P:GO:0008152	P:metabolic process
L21T78P018R01683	---NA---	701	6	1.0E-3.96311E-53	71.0%	1	P:GO:0044408	P:growth or development of symbiont on or near host surface
L19T52P001R00233	d-lactate dehydrogenasemitochondrial precursor	719	20	1.0E-3.36019E-79	82.4%	5	P:GO:0006468<br>F:GO:0016491<br>F:GO:0005524<br>F:GO:0050660<br>F:GO:0004674	P:protein amino acid phosphorylation<br>F:oxidoreductase activity<br>F:ATP binding<br>F:FAD binding<br>F:protein serine/threonine kinase activity
L10T34P076R07115	pyrimidine 5-	699	20	1.0E-4.65982E-70	82.05%	2	F:GO:0003824<br>P:GO:0008152	F:catalytic activity<br>P:metabolic process
L07T11P036R03403	mitochondrial carrier protein	704	20	1.0E-2.41085E-74	81.15%	5	C:GO:0005743<br>C:GO:0016021<br>F:GO:0005488<br>P:GO:0006810<br>F:GO:0005215	C:mitochondrial inner membrane<br>C:integral to membrane<br>F:binding<br>P:transport<br>F:transporter activity
L06T34P032R03057	dimethylaniline monooxygenase	687	20	1.0E-3.66393E-72	67.05%	1	F:GO:0016491	F:oxidoreductase activity

L02T34P066R06130	translationally-controlled tumor protein	771	20	1.0E-1.37999E-68	79.6%	6	C:GO:0005840<br>P:GO:0006979<br>P:GO:0006412<br>C:GO:0005829<br>C:GO:0005739<br>C:GO:0005874	C:ribosome<br>P:response to oxidative stress<br>P:translation<br>C:cytosol<br>C:mitochondrion<br>C:microtubule
T59C36	4-hydroxyphenylpyruvate dioxygenase	658	20	1.0E-1.72259E-60	76.8%	4	P:GO:0006572<br>F:GO:0005506<br>F:GO:0003868<br>P:GO:0006559	P:tyrosine catabolic process<br>F:iron ion binding<br>F:4-hydroxyphenylpyruvate dioxygenase activity<br>P:L-phenylalanine catabolic process
T34C764	Fusarium oxysporum trypsin at atomic resolution	1234	20	1.0E-1.1045E-123	71.85%	2	P:GO:0006508<br>F:GO:0004252	P:proteolysis<br>F:serine-type endopeptidase activity
T34C669	Fusarium oxysporum trypsin at atomic resolution	743	20	1.0E-1.8322E-115	69.3%	2	P:GO:0006508<br>F:GO:0004252	P:proteolysis<br>F:serine-type endopeptidase activity
L92S34P003R00259	phosphoketolase	574	20	1.0E-2.57179E-66	80.9%	2	F:GO:0016829<br>P:GO:0008152	F:lyase activity<br>P:metabolic process
L19T52P002R00673	4-hydroxyphenylpyruvate dioxygenase	577	20	1.0E-3.08543E-67	85.6%	4	P:GO:0006572<br>F:GO:0005506<br>F:GO:0003868<br>P:GO:0006559	P:tyrosine catabolic process<br>F:iron ion binding<br>F:4-hydroxyphenylpyruvate dioxygenase activity<br>P:L-phenylalanine catabolic process
L10T34P076R07113	lipoprotein aminopeptidase lpql	715	20	1.0E-9.38267E-48	59.85%	1	F:GO:0016787	F:hydrolase activity
L03T34P074R06985	transcription factor	636	18	1.0E-1.21354E-68	56.77%	1	F:GO:0005488	F:binding
L03T34P070R06561	sphingomyelin phosphodiesterase	614	20	1.0E-9.77945E-65	67.2%	2	P:GO:0044408<br>F:GO:0016787	P:growth or development of symbiont on or near host surface<br>F:hydrolase activity
L02T34P100R09404	endochitinase 42	624	20	1.0E-7.40054E-108	97.45%	5	F:GO:0004568<br>P:GO:0006032<br>F:GO:0043169<br>F:GO:0008061	F:chitinase activity<br>P:chitin catabolic process<br>F:cation binding<br>F:chitin binding

							C:GO:0005576	C:extracellular region
L02T34P012R01078	snf7 family protein	654	20	1.0E-1.0455E-17	78.75%	2	P:GO:0015031<br>P:GO:0043581	P:protein transport<br>P:mycelium development
T34C36	rta1 domain	1101	20	1.0E-2.59568E-57	63.4%	1	P:GO:0050896	P:response to stimulus
L10T34P102R09645	mitochondrial ornithine carrier protein	662	20	1.0E-1.93466E-43	73.45%	4	C:GO:0016021<br>C:GO:0005739<br>F:GO:0005488<br>P:GO:0006810	C:integral to membrane<br>C:mitochondrion<br>F:binding<br>P:transport
L08T34P063R05860	formyltetrahydrofolate deformylase	752	20	1.0E-3.44933E-93	82.3%	7	F:GO:0008864<br>P:GO:0043581<br>P:GO:0006189<br>F:GO:0016742<br>F:GO:0016597<br>F:GO:0003984<br>P:GO:0009082	F:formyltetrahydrofolate deformylase activity<br>P:mycelium development<br>P:'de novo' IMP biosynthetic process<br>F:hydroxymethyl-, formyl- and related transferase activity<br>F:amino acid binding<br>F:acetolactate synthase activity<br>P:branched chain family amino acid biosynthetic process
