# Supplementary material for: Gene expression analysis of the biocontrol fungus Trichoderma harzianum in the presence of tomato plants, chitin, or glucose using a high-density oligonucleotide microarray
Source: BMC Microbiol. 2009 Oct 13;9:217. doi: 10.1186/1471-2180-9-217 (PMC2768740; doi:10.1186/1471-2180-9-217)
Supplement: Additional file 5 — Table S5. Genes induced in T. harzianum in contact with tomato plant roots. [file 1471-2180-9-217-S5.PDF]

Table S5. Genes induced in T. harzianum in contact with tomato plant roots.

Sequence ID[a, c, d]	Hit Description[e]	GO-Description[f]	Fold[g]		
			MS-P	MS-Ch	MS-G
Carbohydrate, lipid, amino acid and nucleotide metabolism					
T34C721	o-glycosyl hydrolase family 2	P:carbohydrate metabolic process; P:growth or development of symbiont on or near host surface	2.7	1.4	0.6
L52T3KP007R00584	hypothetical O-glycosyl hydrolase	P:carbohydrate metabolic process	2.0	1.1	3.1
L11T34P099R09379 (2)	aldose 1-epimerase	P:carbohydrate metabolic process; P:growth or development of symbiont on or near host surface	2.6	0.9	0.0
L92S34P003R00259	phosphoketolase	P:carbohydrate metabolic process; F:lyase activity	2.2	2.5	1.4
L52T3KP015R01357	d-lactate dehydrogenase	P:carbohydrate metabolic process; P:mycelium development	2.3	1.3	1.2
L19T52P001R00233	d-lactate dehydrogenase mitochondrial precursor	F:oxidoreductase activity; F:FAD binding	2.8	1.7	6.0
L92S34P005R00458 (2)	glucan synthase	P:1.3-beta-glucan biosynthetic process	2.8	1.0	0.3
L92S34P002R00160	phosphatidylserine synthase	P:phospholipid biosynthetic process; P:mycelium development	2.0	1.2	0.4
L52T3KP019R01759	dihydroxyacetone kinase	P:glycerol metabolic process ; P:growth or development of symbiont on or near host surface	2.1	1.0	2.0
L03T34P070R06561	acid sphingomyelin phosphodiesterase	F:hydrolase activity; P:growth or development of symbiont on or near host surface	2.8	8.2	1.2
T34C611	acetylornithine aminotransferase	P: Arginine metabolic process. P:amino acid biosynthetic process	2.4	1.5	14.6
L19T52P002R00673 (2)	4-hydroxyphenylpyruvate dioxygenase	P:tyrosine catabolic process; P:L-phenylalanine catabolic process	2.2	5.1	1.7
L10T34P076R07115	pyrimidine 5'-nucleotidase	P:metabolic process; F:catabolic activity	3.0	0.9	16.7
Energy metabolism					
L03T34P070R06566 (9)	mitochondrial ATPase inhibitor	F:enzyme inhibitor activity	2.7	1.8	0.7
L52T3KP010R00917	nadh:ubiquinone oxidoreductase (complex I lyr family)	F:oxidoreductase activity acting on NADH or NADPH; C:mitochondrial inner membrane	2.0	1.1	2.5
Vitamin and cofactor metabolism					
L51TP1P003R00220	gtp cyclohydrolase I	P:tetrahydrobiopterin biosynthetic process; P:growth or development of symbiont on or near host surface	2.1	1.1	1.6
L08T34P063R05860	formyltetrahydrofolate deformylase	P:'de novo' IMP biosynthetic process; P:mycelium development; F:hydroxymethyl-, formyl- and related transferase activity	2.1	2.2	36.3
Detoxification					
estExt_fgenesh5_pg.C_220056[b]	2-nitropropane dioxygenase	F:oxidoreductase activity	2.4	1.3	1.4
L06T34P032R03057	dimethylaniline monooxygenase	F:oxidoreductase activity	2.3	0.7	45.5
T34C36	rta1 domain protein	P:response to stimulus	4.5	3.1	12.6
TSTC134	glutathione-dependent formaldehyde-activating enzyme	P:growth or development of symbiont on or near host surface; F:carbon-sulphur lyase activity	2.1	1.1	2.0
Signalling, transcription and translation					
L02TP1P006R00499	mFLJ00348 protein	P: I-kappaB kinase/NF-kappaB cascade; F: binding	2.5	1.6	1.9
L02T34P018R01663	transcriptional effector	P:regulation of transcription DNA-dependent	4.1	1.8	1.0
L03T34P074R06985*	transcription factor (PacC)	F:binding	2.6	7.7	0.2
L03T34P059R05502	pentatricopeptide repeat protein	F:FMN binding; P:mycelium development	2.1	1.5	0.1
TSTC120	ribosomal s30/ubiquitin fusion	P:translation	2.4	1.3	2.0

Sequence ID[a, c, d]	Hit Description[e]	GO-Description[f]	Fold[g]		
			MS-P	MS-Ch	MS-G
L53TP1P028R02620	60s ribosomal protein l44	P:translation	2.2	1.2	2.1
L51TP1P002R00166	60s ribosomal protein l40	P:translation	2.3	1.4	1.9
L02T34P066R06130 (2)	translationally-controlled tumour protein	P:translation	2.1	1.7	2.8
Posttranslational modification and protein folding					
L55TSTP004R00316	ubiquitin conjugating enzyme	P:post-translational protein modification	2.0	1.2	1.7
L51TP1P019R01734	prefolding subunit	P:protein folding	2.1	1.3	1.9
L54TP1P031R02884	tpr domain protein	F:heat shock protein binding; P:mycelium development	2.1	1.2	1.2
Transport					
L10T34P102R09645	mitochondrial ornithine carrier protein	P:transport	3.0	2.6	13.6
L07T11P036R03403	mitochondrial carrier protein	P:transport	2.1	1.6	2.1
TP1C33	mitochondrial outer membrane protein	P:protein transport	4.1	1.4	1.7
L02T34P012R01078	snf7 family protein	P:protein transport; P:mycelium development	2.7	3.1	0.7
Cytoskeleton and cell wall					
T34C109	profilin-like protein	P:actin cytoskeleton organization and biogenesis	2.2	1.5	5.4
L50TH2P020R01889	dynein heavy chain	P:microtubule-based movement	2.0	1.0	1.1
L20T59P001R00153	hydrophobin	P:hyphal growth	2.1	0.1	0.9
Host interaction					
L03T34P047R04348	epl1 protein	P:interaction with host; P:pathogenesis	2.3	1.5	0.6
L02T34P100R09404*	endochitinase 42	P:chitin catabolic process	5.2	322.4	1.9
T34C669* (2)	trypsin like protease (PRA1)	P:proteolysis	13.7	180.5	0.8
T34C294*	aspartic peptidase (P6281)	P:proteolysis	2.2	0.7	0.0
L10T34P112R10010* (3)	qid74 protein	-	4.4	1.4	1.5
Unknown function					
L07T11P033R03099 (27)	mitochondrial protein of unknown function	C:mitochondrion	2.5	1.0	1.7
L50TH2P008R00685	hypothetical protein	P:growth or development of symbiont on or near host surface	2.0	1.1	1.2
L21T78P018R01683	hypothetical protein	P:growth or development of symbiont on or near host surface	2.4	1.5	4.0

[a] List of gene transcripts (EST-derived or genome-derived) from *Trichoderma* spp. whose probe sets showed significant up-regulation (fold-change > 2.0 and FDR = 0.23) in microarray experiments after hybridization with cDNA from *T. harzianum* CECT 2413 grown for 9 hours in contact with tomato plant roots in MS medium as compared with the control condition in MS medium alone. EMBL accession numbers of the EST collection are given in additional file 6. ESTs that were assembled into contigs are given in additional file 7.

[b] *T. reesei* genome-based transcripts are available at *T. reesei* genome database [38].

[c] Numbers in parenthesis indicate redundancy: number of transcript sequences having the same best BLAST hit.

[d] An asterisk indicates that the transcript sequence matched a *T. harzianum* CECT 2413 known gene.

[e] Based on BLAST hits using an E value < 10^{-5} as indicative of significance.

[f] Biological processes (P), molecular functions (F) and cellular components (C) are based on functional GO categories inferred from electronic annotation through Blast2GO. More information about the annotation results is given in additional file 4.

[g] Relative gene transcript expression level in the presence of tomato plants (MS-P), chitin (MS-Ch) or glucose (MS-G) *vs*. MS medium alone.
